# 

**Published:** 1840-11

**Authors:** 


					﻿THE
WESTERN JOURNAL
OF
MEDICINE AND SURGERY.
COLLABORATORS.
Edward H. Barton, M. D. Profes-
sor of the Theory and Practice of
Medicine, in the Medical College of
Louisiana.
William J. Barbee, M. D. George'
town, Ky.
George W. Bayless, M. D. Demon-
strator of .Anatomy in the Louis-
ville Medical Institute.
A- H. Buchanan, M. D. Columbia,
Tennessee.
Charles Caldwell, M. D. Professor
of the Institutes of Medicine and
Medical Jurisprudence in the Lou-
isville Medical Institute.
Samuel A. Cartwright, M. D. Mis-
sissippi.
A. Clapp, M. D. New Albany, In-
diana.
Jedediaii Cobb, M. D. Professor of
Anatomy in the Louisville Medical
Institute.
John Esten Cooke, M. D. Professor
of the Theory and Practice of Medi-
cine in the Louisville Medical In-
stitute.
Samuel D. Gross, M. D. Professor of
Surgery in the Louisville Medical
Institute.
John Hardin, M. D. Munfordsville,
Ky.
John P. Harrison, M. D. late Profes-
sor of Materia Medica, in the Cin-
cinnati College.
John F. Henry, M. D. Bloomington,
Illinois.
Samuel P. Hildreth, M. D. Marietta,
Ohio.
Samuel Hogg, M. D. Nashville, Ten-
nessee.
M. Z. Kreider, M. D., Lancaster,
Ohio.
Moses L. Linton, M. D. Springfield,
Kentucky.
Henry Miller, M. D. Professor of
Obstetrics and the Diseases of Wo-
men and Children in the Louisville
Medical Institute.
John W. Monette, M. D. Washing-
ton, Mississsippi.
Samuel B. Richardson, M. D., Lec-
turer on Anatomy and Surgery, <^c,
Louisville, Ky.
John L. Riddell, M. D. Professor of
Chemistry in the Medical College
of Louisiana.
Landon C. Rives, M. D. late Pro-
fessor of Obstetrics in the Medical
Department of the Cincinnati Col-
lege.
Charles W. Short, M. D. Professor
of Materia Medica in the Louisville
Medical Institute.
G. Troost, M. D. Professor of Chem-
istry and Mineralogy in the Uni-
versity of Nashville.
Amasa L. Trowbridge, M. D. Pro-
fessor of Surgery, Willoughby Uni-
versity, Ohio.
John A. Warder, M. D. Cincinnati,
Ohio.
William Wood, M. D. Cincinnati,
Ohio.
OCT*Tiie subscription year of the Western Journal
of Medicine and Surgery is now nearly ended, and
we have now a right to call upon subscribers, one and
all, to remit. Money enclosed to us by mail is always
at our risk, but subscribers are expected to procure
the post office frank or to pay postage.
PRENTICE & WEISSINGER.
Louisville, Nov. 2d, 1840.
The Western Journal of Medicine and Surgery is published monthly by
the undersigned, at the corner of Main and Fifth streets, Louisville, at $5 per
annum, payable in advance. Each number contains from 80 to 84 pages making
two volumes in the year of about 500 pages.
Contributions to its pages by the Physicians of the Valley of the Mississippi,
addressed to the Editors, are respectfully solicited.
Letters on business, to be addressed, postage paid, to the publishers.
July 25, 1840.	PRENTICE & WEISSINGER
				

